# Risk factors for the development of systemic sclerosis: a systematic review of the literature

**DOI:** 10.1093/rap/rky041

**Published:** 2018-10-11

**Authors:** Samuel Abbot, David Bossingham, Susanna Proudman, Caroline de Costa, Albert Ho-Huynh

**Affiliations:** 1 College of Medicine & Dentistry, James Cook University, Cairns, Queensland, Australia; 2 Rheumatology Unit, Royal Adelaide Hospital, Adelaide, South Australia, Australia; 3 Discipline of Medicine, University of Adelaide, Adelaide, South Australia, Australia

**Keywords:** scleroderma, systemic sclerosis, risk factors, epidemiology

## Abstract

**Objectives:**

Although numerous studies have investigated the roles of various genetic, epigenetic and environmental factors that may impact its aetiology, SSc is still regarded as an idiopathic disease. Given that there is significant heterogeneity in what has been proposed to influence the development of SSc, this systematic review was conducted to assess the impacts of different factors on the aetiology of scleroderma.

**Methods:**

The search was performed in the PubMed, CINAHL and SCOPUS databases on 17 May 2017. Any study that made explicit reference to scleroderma or SSc that had information about the risk factors or epidemiology of the disease was included. The extracted outcome variables were prevalence, gender preponderance, geographical distribution, family history and various proposed environmental risk factors.

**Results:**

One thousand five hundred and seventy-four articles were screened for eligibility. Thirty-four articles were eligible for the systematic literature review.

**Conclusion:**

Age between 45 and 64 years, female sex, positive family history and exposure to silica were found to be risk factors. There were conflicting findings regarding the impact of exposure to organic solvents and microchimerism. No relationship between infectious agents, alcohol consumption or cigarette smoking and the development of SSc was identified.

**Key messages**

- Middle age, female sex, positive family history and exposure to silica are risk factors for scleroderma.
- Infectious agents, alcohol and cigarette smoking are not risk factors for scleroderma development.

## Introduction

Scleroderma, or SSc, refers to a heterogeneous group of autoimmune fibrosing disorders that are chronic, progressive and can lead to fatal complications [1]. The term scleroderma was first used in 1836 by Giovambattista Fantonetti, who published a case study about a 30-year-old woman with the condition in his journal *Effemeridi della Scienze Mediche* [2]. Fantonetti derived the term from the Greek words skleros and dérma, which respectively translate to hard and skin [3].

The epidemiology of SSc has provided some insights into possible causes of the disease. SSc is a rare disease, which suggests that the genetic and environmental factors that predispose to it are also rare [4]. There is marked geographical variation in the prevalence of SSc, ranging from 7 to 658 per million [5–8]. Although this is likely to be attributable, in part, to discrepancies in disease ascertainment, it might indicate that there are environmental exposures that predispose to SSc in certain parts of the world [9]. Studies agree on a 7:1 female preponderance, and the onset in most patients is between the ages of 30 and 50 years, which suggests that hormonal factors, pregnancy and age-related influences might play a role [9–13]. Paradoxically, there is a 13- to 19-fold increase in the risk of developing SSc if one has a sibling with the disease, although SSc very rarely runs in families [10–12]. Nonetheless, we still know very little about the underlying causes of SSc and its pathogenesis. The aim of this systematic review is to synthesize what has been discovered regarding the risk factors for the development of SSc, in an attempt to enhance our understanding of the causes of this debilitating disease.

## Methods

This study was carried out according to the Preferred Reporting Items for Systematic Reviews and Meta-Analysis (PRISMA) guidelines and is registered at the International Prospective Register of Systematic Reviews [14, 15]. The registration number for this review on the International Prospective Register of Systematic Reviews website is CRD42017067304, and a published protocol for this study can be found there.

### Eligibility criteria

In order to be included in this systematic review, the articles had to be studies written in English that involved humans and made explicit reference to scleroderma or SSc. There was no restriction on publication date or study type. Articles were excluded if they used the same data published in another study, in order to reduce the effect of publication bias. The studies had to examine adult-onset scleroderma, rather than juvenile scleroderma (which is a distinct entity from SSc and may have a different aetiology). Articles were excluded if they focused on the proposed effects of silicone breast implants on SSc aetiology, because many studies have found no relationship [4, 6, 7, 13, 16–18].

### Search strategy

The systematic search was done on 17 May 2017, using the following databases: PubMed, CINAHL and SCOPUS. The search strategy for PubMed was constructed by the first author, and was as follows:

(((risk factors OR risk factor OR risk OR probability OR epidemiolog* OR aetiolog* OR etiolog*)) AND (SSc OR scleroderma)) AND (develop*).

The search strategy was modified to fit CINAHL and SCOPUS. Duplicates among the databases were excluded. The titles and abstracts of the articles were then screened independently by two authors (S.A. and A.H.-H.). If the title and/or abstract met the eligibility criteria, full texts of the articles were obtained. Any discrepancies in the selection process between the two authors were resolved by discussion. Three additional studies were identified from reference lists of the included articles [9, 10, 19].

### Data collection and data items

The articles included in the systematic review were analysed for risk factors that have been suggested for the subsequent development of SSc; these were: patient demographics; positive family history for SSc; occupational exposures to noxious substances (e.g. silica); exposure to infectious agents; microchimerism, oestrogen and pregnancy-related events; cigarette smoking and alcohol consumption; low birthweight and small-for-gestational age; and vitamin D exposure.

The study data collected from the articles were study type, sample size, methodology, author, country and year of publication.

### Risk of bias within studies and quality assessment

The quality of each of the articles was assessed according to their study type. A modified version of the Newcastle–Ottawa Scale was used to assess the quality and risk of bias within the case–control and cohort studies [20]. The Newcastle–Ottawa Scale grades articles according to their case selection, comparability and ascertainment of exposure. For systematic reviews and meta-analyses, a standardized quality assessment scale constructed by the National Heart, Lung and Blood Institute was used, and for narrative reviews, a scale proposed by La Torre *et al.* [21, 22], called the International Narrative Systematic Assessment score, was used.

## Results

### Study selection

**Fig. 1:**
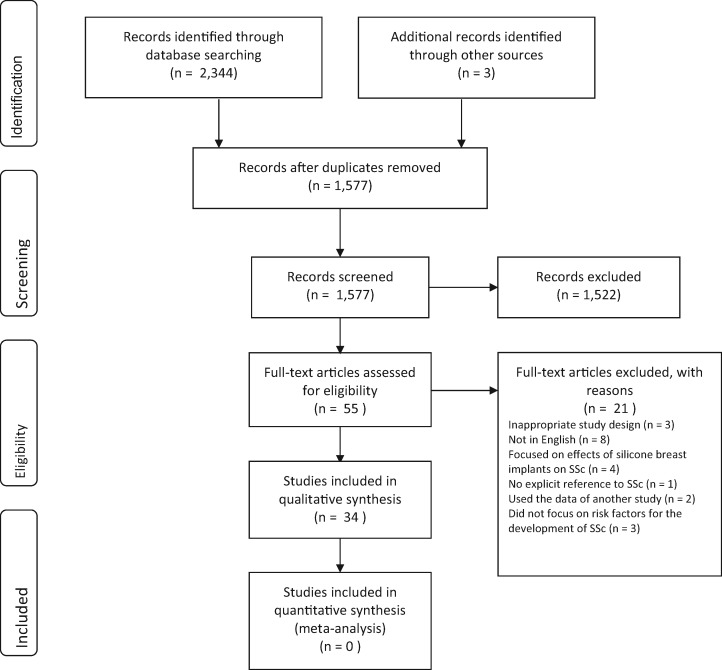
PRISMA flow diagram depicting the process by which articles were screened PRISMA: preferred reporting items for systematic reviews and meta-analysis.

The search retrieved a total of 2347 results. Seven hundred and seventy duplicates were identified; therefore, 1577 titles were screened. Of these articles, 1268 were excluded because of an irrelevant title, and a further 254 were rejected when the abstract was deemed irrelevant. Fifty-five articles underwent full-text review, which included 5 meta-analyses, 2 systematic reviews, 27 narrative reviews, 15 case–control studies, 3 cohort studies and 3 case reports. Three articles were excluded because they had an inappropriate study design [23–25]. A further eight were excluded because they were not written in English [26–33]. Four articles were excluded because of their focus on the proposed effects of silicone breast implants on SSc aetiology [7, 16, 34, 35]. Another two articles were excluded because they used the data of another study [19, 36]. One article was excluded because it did not make specific reference to SSc [37]. Finally, three articles were excluded because they did not focus on risk factors that lead to the development of SSc [38–41]. A total of 34 articles were finally included in the systematic review [4–6, 8–10, 12, 13, 17, 18, 41–64]. These are outlined in the PRISMA flow diagram shown in Fig. 1. Upon analysis of the included texts, the methods, risk factors examined and outcomes of each text were noted, and these are listed in Table 1.

**Table 1:** Characteristics of the studies examined in the systematic review

Author, year and country	Type of study and methods	Risk factor(s) examined and ascertainment of exposure	Outcomes
Bilgin *et al.* 2015 Turkey [44]	Case–control (April 2009–March 2012). 30 SSc patients who were admitted to the Konya Training and Research Hospital *vs* 30 age- and sex-matched controls	Exposure to infectious agents. ELISAs used to detect antibodies against various bacteria and viruses in subjects’ sera	A higher prevalence of infectious agents was found in the SSc patients than in the healthy controls (e.g. 73.3% of the SSc patients had *Helicobacter pylori* antibodies *vs* 46.6% in the control group)
Burns *et al.* 1996 USA [17]	Case–control (1985–1991). 274 female SSc cases in Michigan and 1184 female population-based controls matched by race, age and geographical region	Exposure to silica. Telephone interview	No significant effect found among those who worked with or around silica (adjusted OR: 1.5; 95% CI: 0.76, 2.93)
Chaudhary *et al.* 2011 USA [46]	Case–control (1998–2009). 621 SSc patients enrolled in the GENISOS or Scleroderma Family Register Studies were matched 2:1 by age, sex, ethnicity and state of residence to controls	Cigarette smoking. Smoking history was ascertained via chart review of the BRFSS and via telephone interview	Cigarette smoking was not found to be a risk factor for SSc (*P* = 0.842; OR: 1.020; 95% CI: 0.839, 1.240).
Cockrill *et al.* 2010 USA [48]	Case–control (case–sibling design) (1998–2009). 987 SSc patients enrolled in the GENISOS or Scleroderma Family Register Studies were matched with their unaffected sibling controls (*n* = 3088)	Increasing birth order, gravidity and parity. Data were obtained from the Scleroderma Family Registry and DNA Repository	Risk of SSc increased with increasing birth order (OR: 1.25, 95% CI: 1.06, 1.50 for birth order 2–5; OR: 2.22, 95% CI: 1.57, 3.15 for birth order 6–9; and OR: 3.53, 95% CI: 1.68, 7.45 for birth order 10–15). History of one or more pregnancies was found to be a risk factor for SSc (OR: 2.8). History of one or more pregnancy losses without any live births had the strongest association with SSc (OR: 9.56, 95% CI: 2.12, 43.15)
Donzelli *et al.* 2015 Italy [50]	Case–control (June 2012–November 2013). 332 SSc cases were identified from the rheumatological outpatient clinics of four Italian hospitals. These cases were matched by age and sex to 243 controls from the surgical outpatient clinic of a hospital in Florence	Low birth weight and small-for-gestational age. A questionnaire and an interview were used to collect self-reported perinatal information on the subjects	Low birth weight increases risk of SSc (OR: 2.59; 95% CI: 1.39, 5.05)
			Small-for-gestational age increases risk of SSc (OR: 3.93; 95% CI: 1.92, 8.07)
Garabrant *et al.* 2003 USA [51]	Case–control (1980–1992). 660 female SSc cases were identified from numerous databases and mailing lists in Michigan and Ohio. These were matched to 2227 female controls, who were chosen by random digit dialling telephone sampling	Exposure to solvents. Subjects were interviewed by telephone. An expert then verified the exposures with a retrospective exposure assessment	Paint thinners and removers were associated with SSc (OR: 2.0, 95% CI: 1.5, 2.6)
			Other petroleum distillates and specific solvents (e.g. trichloroethylene) were not significantly associated with SSc
Kütting *et al.* 2006 Germany [54]	Case–control. 109 SSc patients who were part of a SSc support group were matched to 66 MS patients who were part of a MS support group	Exposure to solvents. Subjects were sent a questionnaire and asked to return it anonymously	A non-significant association between occupational exposure to solvents and risk of SSc was found for the male subgroup (OR: 4.794; 95% CI: 0.459, 69.901). No such association found for the other subgroups
Marie *et al.* 2017 France [55]	Case–control (2005–2008). 100 SSc cases who were seen in three French medical centres were matched by age, sex and smoking habit to 300 controls	Exposure to heavy metals. Subjects underwent detection of heavy metal traces in their hair samples, using ICP-MS	Significant associations with SSc were found for palladium, cadmium, zinc, antimony, mercury and molybdenum
Marie *et al.* 2014 France [56]	Case–control (2005–2008). 100 SSc cases who were seen in three French medical centres were matched by age, sex and smoking habit to 300 controls	Exposure to silica and solvents. Subjects were interviewed using a questionnaire, then a committee retrospectively evaluated exposure	Association found for silica (OR: 5.32; 95% CI: 2.25, 13.09) and for some specific solvents, but not for ‘any type of solvent’ (OR: 1.59; 95% CI: 0.93, 2.67)
Nietert *et al.* 1999 USA [60]	Case–control (March 1995–February 1997). 178 SSc cases diagnosed at the Medical University of South Carolina and 200 controls with musculoskeletal disorders	Exposure to solvents. Questionnaire regarding occupation and hobbies, and ELISA of blood samples for Scl70 detection	Overall participation in SOH was not associated with SSc. Odds of having both SOH and occupational exposure were much greater among those positive for Scl70 compared with controls (OR: 5.8; 95% CI: 1.9, 17.7)
Pisa *et al.* 2001 Italy [61]	Case–control (January 1997–June 1999). 46 female SSc cases diagnosed at an Italian hospital were frequency matched to 153 female controls with orthopaedic disorders	Gravidity and parity. Interview and questionnaire	Parous women had reduced risk of SSc (OR: 0.3; 95% CI: 0.1, 0.8). The risk decreased with increasing number of children. Abortive pregnancies were inversely related to SSc risk (OR: 0.5; 95% CI: 0.2, 1.5)
Roberts-Thomson *et al.* 2006 Australia [9]	Case–control (1993–2002). 353 cases and controls obtained from the Australian Bureau of Statistics consensus	Sex, family history, geographical distribution, ethnicity	Female:male ratio approximated 5:1. Family history was the strongest risk factor (OR: 14.3; 95% CI: 5.9, 34.5). 2.5-fold increased risk for subjects born in continental Europe. Ethnicity did not seem to be a risk factor
Russo *et al.* 2014 Australia [63]	Case–control (1993–2013). 387 SSc cases enrolled in the South Australian Scleroderma Register and 457 controls who were either patients or employees of the authors’ hospitals	Increasing birth order, gravidity and parity. Questionnaire	No significant relationship was found for either birth order or family size with SSc. SSc patients were more likely to be multiparous than controls (OR = 1.8; 95% CI: 1.1, 2.98)
Şahin *et al.* 2013 Turkey [64]	Case–control. 80 female SSc patients and 40 healthy female controls	Microchimerism and parity. Questionnaire regarding subjects’ pregnancy history and PCR of peripheral blood samples to detect microchimerism	SSc was more prevalent in women who had given birth and even more common in those who had male children (*P* <0.05). Microchimerism was more common in SSc group than controls (*P* = 0.180)
Aryal *et al.* 2001 USA [42]	Systematic review and meta-analysis of eight articles published between 1989 and 1998	Exposure to solvents. Synthesized the findings of the eight included studies	Organic solvents were found to be a risk factor for SSc (RR: 2.91; 95% CI: 1.60, 6.00)
Barragán-Martínez *et al.* 2012 Colombia [43]	Systematic review and meta-analysis of 33 articles published between 1982 and 2011	Exposure to solvents. Synthesized the findings of eight of the included studies	Organic solvents were found to be a risk factor for SSc (OR: 2.54; 95% CI: 1.23, 5.14)
McCormic *et al.* 2010 USA [58]	Systematic review and meta-analysis of 16 articles published between 1967 and 2007	Exposure to silica. Synthesized the findings of the 16 included studies	Significant heterogeneity was detected (*I*^2^ = 97.2%; *P *<0.01). CERR = 3.20 (95% CI: 1.89, 5.43). CERR for females = 1.03 (95% CI: 0.74, 1.44). CERR for males = 3.02 (95% CI: 1.24, 7.35)
Zhao *et al.* 2016 China [5]	Systematic review and meta-analysis of 14 case–control studies published between 1989 and 2014	Exposure to solvents	Organic solvents increase risk of SSc (OR: 2.07; 95% CI: 1.55, 2.78). RR was higher in men (OR: 5.28; 95% CI: 3.46, 8.05) than women (OR: 1.62; 95% CI: 1.34, 1.96). Trichloroethylene exposure increases risk of SSc (OR: 2.07; 95% CI: 1.34, 3.17)
Antico *et al.* 2012 Italy [41]	Systematic review of 219 articles published between 1973 and 2011	Lack of vitamin D. Synthesized the findings of seven of the included studies	Four studies out of seven detected lower levels of vitamin D in SSc patients than controls (46–84 *vs* 40%)
Radić *et al.* 2010 Croatia [62]	Systematic review of 52 articles published between 1984 and 2008	*Helicobacter pylori* infection	Supports hypothesis of *H. pylori* infection being a cofactor in SSc aetiology
Allanore *et al.* 2015 USA [8]	Narrative review of 208 articles published between 1980 and 2015	Geographical distribution, family history, smoking, alcohol, silica, vinyl chloride, solvents, infectious agents	SSc is more common in southern Europe, the USA and Australia than the UK and Asia. RR of first-degree relatives = 13. Smoking and alcohol not associated. Silica, vinyl chloride, organic solvents and infectious agents ‘might be involved’
Brasington *et al.* 1991 USA [45]	Narrative review of 15 articles published between 1965 and 1989	Solvents	Exposure to organic solvents is a risk factor for SSc
Chen *et al.* 2003 Australia [47]	Narrative review of 80 articles published between 1971 and 2001	Age, family history, silica, solvents, microchimerism	SSc onset is most common between ages of 30–50 years. Positive family history is the strongest risk factor for SSc (RR = 10–16). Silica and organic solvents are a risk factor for SSc. Microchimerism may be only one part in a multifactorial pathogenesis of SSc
De Martinis *et al.* 2016 Italy [49]	Narrative review of 93 articles published between 1957 and 2015	Geographical distribution, silica, solvents, infectious agents, smoking	SSc is more common in southern Europe, the USA and Australia than the UK and Asia. Current evidence supports silica as a risk factor, is ambiguous regarding organic solvents and infectious agents, and does not support smoking as a risk factor
Dospinescu *et al.* 2013 UK [6]	Narrative review of 38 articles published between 1914 and 2012	Silica, solvents, smoking	Current evidence suggests silica is a risk factor for SSc, solvents may or may not be a risk factor, and smoking is not a risk factor
Gaubitz 2006 Germany [52]	Narrative review of 12 articles published between 1988 and 2003	Geographical distribution, silica, solvents, vinyl chloride	SSc is more common in the USA and Australia than the UK. Silica, organic solvents and vinyl chloride significantly increase risk of SSc
Hamamdzic *et al.* 2002 USA [53]	Narrative review of 34 articles published between 1984 and 2001	Infectious agents, microchimerism	Development of SSc is unlikely to depend exclusively on an infectious agent, but rather as a result of the interactions between the infectious agent and a cascade of host-specific factors and events. Microchimeric cells are more common in women with SSc than in healthy controls
Marie *et al.* 2015 France [18]	Narrative review of 121 articles published between 1914 and 2015	Silica, solvents, smoking, infectious agents, vinyl chlorides, epoxy resins	There is a marked correlation between SSc onset and exposure to silica and various organic solvents. Insufficient data to suggest that infections, smoking, physical agents, vinyl chlorides and epoxy resigns play a causative role in the development of SSc
Mayes 1996 USA [12]	Narrative review of 56 articles published between 1960 and 1996	Sex, geographical distribution, ethnicity, family history	SSc occurs much more frequently in women than in men. SSc is five times more common in the USA than in Britain and Japan. African-American patients have a higher age-specific incidence rate and more severe disease than Caucasians. More than 99% of SSc patients report no first-degree relatives who have SSc
Mayes 1999 USA [57]	Narrative review of 65 articles published between 1967 and 1998	Oestrogens, silica, solvents	Oestrogen replacement therapy increases risk (1.5- to 3-fold), but COCP does not. Silica exposure does not explain most cases of SSc in men and does not play a significant role in women. Role of solvent exposure is unclear
Mora 2009 Argentina [59]	Narrative review of 154 articles published between 1949 and 2007	Silica, vinyl chloride, solvents, infectious agents, microchimerism, oestrogens	Silica exposure is a risk factor. Ambiguous regarding vinyl chloride and organic solvents as risk factors. SSc is associated with *H. pylori* and viral infection. Microchimerism may explain female preponderance. Oestrogen replacement therapy increases risk, but COCP does not
Nikpour *et al.* 2010 Australia [10]	Narrative review of 100 articles published between 1957 and 2010	Geographical distribution, ethnicity, family history, sex, solvents, silica	SSc is more common in the USA and Australia than Japan and Europe. More common in black than white Americans. 13- to 14-fold increased risk if first-degree relative with SSc. 7:1 female preponderance. Solvents and silica are a risk factor for SSc
Silman 1991 UK [13]	Narrative review of 112 articles published between 1914 and 1991	Geographical distribution, genetics, silica, solvents, smoking, alcohol	Prevalence in Japan is much lower than in Western countries. Unlikely that genetic factors are a major cause. Silica exposure is a risk factor. Numerous organic solvents have been implicated. Smoking is not a risk factor, but alcohol is
Silman *et al.* 1994 UK [4]	Narrative review of 39 articles published between 1985 and 1994	Family history, occupational exposures, infectious agents	Multiple affected kinships are rare. Silica and organic solvents may lead to sporadic cases, but the attributable risk is low. There is very little evidence for a viral link to SSc

BRFSS: behavioural risk factor surveillance system; CERR: Combined Estimated Relative Risk; COCP: Combined Oral Contraceptive Pill; ICP-MS: Inductively coupled plasma mass spectrometry; MS: multiple sclerosis; OR: odds ratio; SOH: solvent-orientated hobbies; RR: Relative Risk (of developing SSc with being exposed vs non-exposed to organic solvents).

### Risk of bias across studies

Supplementary Tables S1 and S2, available at *Rheumatology* online, depict the quality assessment and risk of bias of the included articles. The International Narrative Systematic Assessment scoring system that was used to assess the quality of the narrative reviews considered factors such as the objective of the review, selection of studies and presentation of results. A score of 5/7 or higher is considered to be good, and all of the included reviews scored a grade of 5 or higher. Both systematic reviews struggled to fulfil the criteria of the quality assessment scale that was used in this review. They are, however, comprehensive articles that form a coherent argument and do not represent any conflict of interest or publication bias and were thus deemed to be eligible for inclusion. The quality scale used for the meta-analyses was a modified version of the scale used for the assessment of the systematic reviews, which included an additional criterion regarding whether heterogeneity was assessed [21].

**Table 2:** Summary finding of risk factors for the development of SSc

Risk factor	Number of studies	Regarded as a risk factor (based on available evidence)	Level of evidence (based on heterogeneity and quality assessment scale of the included studies)
Female sex	7	Yes	High
Age between 45 and 64 years	4	Yes	High
Geographical distribution and ethnicity	9	Yes	Moderate
Positive family history	7	Yes	High
Exposure to organic solvents	18	Yes	Moderate
Exposure to silica	14	Yes	Moderate
Infections	8	No	Moderate
Insufficient vitamin D exposure	2	Uncertain	Low
Smoking and alcohol	6	No	High
Exposure to heavy metals	1	Yes	Moderate
Exposure to physical agents	1	No	Moderate
Microchimerism	8	Uncertain	Moderate
Low birthweight	1	Yes	Moderate

### Risk factors for the development of scleroderma

A summary of the findings for risk factors for SSc derived from the studies is shown in Table 2.

#### Female sex

Female sex as a risk factor for SSc was assessed in seven of the studies, all of which made note of the marked female preponderance, with female:male prevalence ratios ranging from 3:1 to 8:1 [4, 8, 9, 12, 13, 53].

#### Age

Patient age was investigated as a risk factor in four of the studies [5, 8, 13, 47]. The common finding between all of the studies was that the risk of SSc peaks between the ages of 45 and 64 years [9, 10, 12].

#### Geographical and ethnic group distribution

Nine of the studies examined the effects of geographical location and ethnicity [5, 8–10, 12, 13, 47, 49, 52]. Seven of these articles discovered a higher prevalence in Australia and North America compared with Continental Europe, the UK and Japan [5, 8, 10, 12, 13, 49, 52]. SSc was found to be more prevalent in African-Americans than Caucasian Americans in six of the studies [8, 12, 13, 36, 49, 52]. One study, on the contrary, did not find an association with ethnicity [9]. A north–south gradient in Europe was reported in two of the studies, with the incidence being lower in northern Europe [10, 49].

#### Positive family history

Seven of the studies discussed the impact of a positive family history on SSc susceptibility [4, 8–10, 12, 13, 47]. All of these studies reported a positive family history as a risk factor for SSc, and four of them suggested that it is the strongest risk factor for development of SSc, with odds ratios (ORs) varying between 10 and 16 [8–10, 47]. In contrast, the other three studies argued that it is unlikely that genetic factors are a major cause of SSc [4, 12, 13].

#### Exposure to organic solvents

Occupational exposure to organic solvents was investigated in 18 of the studies, with significantly variable results [5, 6, 8, 10, 13, 18, 42, 43, 45, 47, 49, 51, 52, 54, 56, 57, 59, 60]. Eleven of these articles found a significant correlation between exposure to organic solvents and SSc, with ORs ranging between 2.07 and 2.91 [5, 10, 13, 18, 42, 43, 47, 49, 52, 56, 60]. Conversely, two other studies found that exposure confers a significantly increased risk of SSc to men, but not to women [54, 59]. A further three studies were inconclusive [6, 45, 57]. One article could only find a relationship between paint thinners and removers with SSc, but could not find a significant association between other specified solvents and SSc [50]. Finally, the remaining article argued against any such risk [8].

#### Exposure to silica

Exposure to silica as a risk factor for SSc was examined in 14 of the included articles and was reported to be a significant risk factor in nine of them (including one meta-analysis), with ORs ranging between 3.20 and 25 [6, 10, 13, 18, 47, 49, 52, 56, 58]. In contrast, two articles concluded that occupational exposure to silica may be a significant risk factor for men, but not for women [57, 59]. The final three articles did not find a significant correlation between exposure to silica and SSc [4, 8, 17].

#### Infections

Exposure to infections as a risk factor was studied in eight of the included articles, again with considerable heterogeneity [4, 8, 18, 44, 53, 59, 62]. Four of the articles reported a positive correlation between infectious agents with SSc [8, 44, 59, 62]. In contrast, one of the studies reported that there have been conflicting results in the literature regarding infections and SSc but concluded that their involvement in the disease cannot be ruled out [53]. The final three studies stated that there is insufficient evidence in the literature to implicate either bacterial or viral infection as a risk factor for SSc [4, 18, 49].

#### Insufficient vitamin D exposure

Two of the included studies investigated the effects of hypovitaminosis D on SSc, and they both concurred that vitamin D deficiency can be a risk factor for SSc [41, 49].

#### Cigarette smoking and alcohol consumption

All of the six studies that investigated smoking and alcohol consumption as a risk factor for SSc found neither of them to be significant risk factors [6, 8, 13, 18, 46, 49].

#### Exposure to heavy metals

Exposure to heavy metals was examined in one study, which found a significant correlation between SSc and exposure to a number of heavy metals, including palladium, cadmium, zinc and antimony [55].

#### Microchimerism and pregnancy-related events

Microchimerism refers to the persistence of the retained cells from the fetus of a previous pregnancy in a mother’s peripheral bloodstream. The possible effects of microchimerism as a risk factor for SSc were investigated in eight of the articles included in this review [12, 47, 48, 53, 59, 61, 63, 64]. Five of these articles reported microchimerism as being more common in SSc patients than in controls and found a positive relationship between SSc and a history of pregnancy (especially a history of having had a son) [47, 48, 53, 59, 64]. The other three studies failed to show a relationship and argued against microchimerism as a risk factor [12, 61, 63].

#### Low birthweight

One of the included studies examined low birthweight as a risk factor and found a statistically significant correlation between SSc and low birthweight (OR: 3.93; 95% CI: 1.92–8.07), and between SSc and small-for-gestational age (OR: 2.58; 95% CI: 1.28–5.19) [50]. Table 2 summarizes these findings.

## Discussion

This review found complete concordance with the fact that SSc is primarily a disease of middle-aged women, as expected. Likewise, there was strong concordance regarding the geographical distribution of patients with SSc. In contrast, the finding of a north–south gradient across Europe, with SSc being less frequent in northern Europe, is out of keeping with the findings of an Italian systematic review by Antico *et al.* [10, 41, 49], which found that patients with SSc have lower vitamin D levels than healthy controls, in percentages varying from 46 to 84%. However, this finding was based on a modest total of 313 subjects, whereas the epidemiological studies that have noted the higher frequency of SSc in southern Europe have been based on significantly larger, better-powered registries. Therefore, even if vitamin D deficiency is a risk factor for SSc, it seems that there must be other environmental exposures in southern Europe that predispose to SSc that can still contribute to the development of SSc in patients with normal vitamin D levels.

A positive family history of SSc has repeatedly been reported as the strongest risk factor for the development of SSc, but in spite of this, numerous studies have noted that monozygotic twins with SSc are very rare and that 98% of SSc patients do not have another affected family member of any degree of blood relation [9, 12, 47]. The logical explanation for this would be that there is a modest genetic predisposition for SSc and that the environmental exposures necessary for the development of SSc in a predisposed person are uncommon. Therefore, although the brother of a patient with SSc may be at a 14-fold increased risk of developing SSc throughout his lifetime, the absolute risk of him developing SSc is low, owing to the rarity of the putative environmental exposures necessary for SSc to occur [10].

Perhaps the most contentious issue regarding environmental risk factors for SSc is the exposure to organic solvents. This review included three meta-analyses that examined organic solvents as a risk factor for SSc, all of which found a significant correlation between SSc and exposure to organic solvents, with combined OR values ranging from 2.07 to 2.91 [5, 42, 43]. Although these results appear compelling, there is the possibility of a significant publication bias, either owing to researchers not submitting negative results or owing to journals declining to publish the findings of studies that have not found organic solvents to be a significant risk factor for SSc. Furthermore, all of the four case–control studies included in this review that reported a significant correlation between SSc and organic exposure were retrospectively based on self-reported exposure and were therefore subject to significant recall bias [51, 54, 56, 60]. Finally, as noted by Dospinescu *et al.* [6], exposure to organic solvents has usually been ascertained on the basis of the subjects’ occupations, and it is likely that these subjects (e.g miners) had concomitant, confounding exposure to other environmental agents. Therefore, although the consensus in the literature favours organic solvents as being a significant risk factor for SSc, the evidence for this is still arguably dubious.

Of all of the environmental exposures that have been investigated as risk factors for SSc, occupational exposure to silica appears to be the most convincing. A well-designed meta-analysis by McCormic *et al.* [58] found a significant relationship between occupational silica exposure and SSc, with a combined estimator or relative risk of 3.20 (95% CI: 1.24–7.35). In contrast, a large case–control study by Burns *et al.* [17] did not detect any effect of occupational exposure to silica on the risk of SSc, but this study only included female subjects, who are less likely to experience occupational exposures to silica than men (e.g. as in abrasive grinding, mining and sandblasting). This is in keeping with the finding of Mora *et al.* [59], who argued that occupational exposure to silica significantly increases the risk of SSc in men, but not in women, primarily because the exposure to silica of the male cohort is far greater.

Although the theory of infection leading to molecular mimicry and causing autoimmune disease is biologically plausible, there is currently insufficient evidence to support the notion of infection being a significant risk factor for SSc. In a case–control study involving a modest total of 30 patients with SSc, Bilgin *et al.* [44] found that a higher proportion of SSc patients had antibodies against *Helicobacter pylori*, CNV, EBV and parvovirus B19. However, that study did not account for the impact of other environmental factors on the development of SSc (i.e. did not adjust the results with multivariate logistic regression), which limits the validity of the conclusions. Owing to the poor quality of this evidence, infections are not regarded as a risk factor for SSc [4, 18]. Likewise, there is insufficient evidence for cigarette smoking as a risk factor for the development of SSc, although it might exacerbate the severity and prognosis, once the disease process has begun [6, 46, 49].

Microchimerism as a risk factor for SSc is an interesting theory, which stemmed from the observation of an obvious female preponderance, with females being affected preferentially in the post-childbearing years [47]. It has since been discovered that microchimeric cells are more commonly detected in women with SSc than in healthy controls [47, 53, 59]. These observations motivated many of the studies included in this review, which yielded widely conflicting results. Firstly, Cockrill *et al.* [48] found, in their highly powered case–control study involving 987 patients with SSc and 3088 sibling controls, that a history of one or more pregnancies increased the risk of SSc by an OR of 2.8. In direct contrast to this, Pisa *et al.* [61], in their case–control study, found that a history of pregnancy reduces the risk of SSc (OR: 0.3; 95% CI: 0.1–0.8). It would seem that microchimerism is only one of a multitude of factors that have been implicated in the pathogenesis of SSc but are yet to be proven aetiological factors. This is evidenced by the observation that microchimerism occurs in many healthy, normal women, and SSc often occurs in nulliparous women [47, 61].

Although the articles that examined exposure to heavy metals and low birthweight in this review were strong studies, there are currently insufficient data in the literature to confirm whether or not these factors predispose to the development of SSc [18, 50, 55]. Further research in these areas would be valuable to determine the role of these agents in SSc pathogenesis.

Ultimately, ascertaining the risk factors that predispose to SSc is an ongoing dilemma for three reasons. Firstly, SSc is a very rare disease, making it difficult to obtain cohorts that are adequately powered to provide meaningful data. Secondly, the majority of environmental exposures that are currently under investigation are often experienced concomitantly, and therefore it may be impossible to attribute the development of SSc to one exposure alone. Thirdly, in order to implicate an exposure in SSc aetiology fully, prospective studies are required, because retrospective studies are subject to significant recall bias. The feasibility of such studies is doubtful, because the likelihood of a subject developing SSc as a result of being exposed to a given agent is extremely low, and there are obvious ethical implications involved with risking the development of such a debilitating disease in a subject. Although we have discovered that occupational exposure to silica is a significant risk factor in SSc, in addition to a number of aforementioned patient demographics, the absolute and attributable risks of these factors to SSc aetiology are clearly low, because the vast majority of individuals with these risk factors do not develop SSc. Unfortunately, performing a meta-analysis for the various risk factors for SSc was not possible for the present review, owing to there being excess heterogeneity among the literature. Therefore, as noted numerous times by Roberts-Thomson *et al.* [9, 19], SSc is likely to result from a number of stochastic events that are impossible to identify, thus SSc may continue to be seen as an idiopathic disease.

## Conclusion

In conclusion, this review found age between 45 and 64 years, female sex, living in the USA and Australia, positive family history and occupational exposure to silica to be significant risk factors for the development of SSc. There were conflicting findings regarding the impact of exposure of organic solvents and microchimerism on the development of SSc. There are currently insufficient data to implicate infectious agents as a risk factor. Alcohol consumption and cigarette smoking were not found to be risk factors. Given that we currently have only empirical treatment for SSc, the possible prevention of this disease is of the utmost importance. The results of this review suggest that industries involving exposure to silica should ensure that their staff use protective measures and have regular health checks to limit the possibility of their occupational exposure leading to the development of SSc. Further research is required to ascertain the role of organic solvents, microchimerism and infectious agents as risk factors for SSc.

*Funding*: No specific funding was received from any bodies in the public, commercial or not-for-profit sectors to carry out the work described in this manuscript.

*Disclosure statement*: The authors have declared no conflicts of interest.

## Supplementary Material

Supplementary Data
