# Supplementary material for: Risk factors for the development of systemic sclerosis: a systematic review of the literature
Source: Rheumatol Adv Pract. 2018 Oct 11;2(2):rky041. doi: 10.1093/rap/rky041 (PMC6649937; doi:10.1093/rap/rky041)
Supplement: Supplementary Data [file rky041_supp.docx]

**SUPPLEMENTARY DATA**

**Supplementary Table 1:** Quality Assessment of the Case-Control Studies using a Modified Version of the Newcastle-Ottawa Scale.

| Study | Selection (/5) | Comparibility (/2) | Outcome/Expousure (/3) | Overall modified NOS Score (/10) |
| --- | --- | --- | --- | --- |
| Bilgin et al 2015 | 3 | 1 | 2 | 6 |
| Burns et al 1996 | 3 | 2 | 1 | 6 |
| Chaudhary et al 2011 | 4 | 2 | 2 | 8 |
| Cockrill et al 2010 | 4 | 2 | 1 | 7 |
| Donzelli et al 2015 | 2 | 2 | 2 | 6 |
| Garabrant et al 2003 | 4 | 2 | 3 | 9 |
| Kütting et al 2006 | 0 | 2 | 2 | 4 |
| Marie et al 2017 | 4 | 2 | 3 | 9 |
| Marie et al 2014 | 4 | 2 | 3 | 9 |
| Nietert et al 1999 | 2 | 2 | 3 | 7 |
| Pisa et al 2002 | 2 | 2 | 2 | 6 |
| Roberts-Thomson et al 2004 | 4 | 2 | 1 | 7 |
| Russo et al 2014 | 3 | 1 | 0 | 4 |
| Sahin et al 2013 | 4 | 2 | 2 | 8 |

NOS: Newcastle-Ottawa Scale

**Supplementary Table 2:** Quality Assessment of the Narrative Reviews, Systematic Reviews and Meta-Analyses

| Study | INSA^a^ Score (narrative reviews) (/7) | NHLBI^b^ Score (systematic reviews) (/7) | NHLBI^b^ Score (meta-analyses) (/8) |
| --- | --- | --- | --- |
| Allanore et al 2015 | 6 |  |  |
| Antico et al. 2012 |  | 3 |  |
| Aryal et al 2001 |  |  | 5 |
| Barragán-Martínez et al 2012 |  |  | 7 |
| Brasington et al 1991 | 6 |  |  |
| Brown et al 2004 | 5 |  |  |
| Chen et al 2003 | 5 |  |  |
| De Martinis et al 2015 | 6 |  |  |
| Dospinescu et al 2013 | 6 |  |  |
| Gaubitz 2006 | 5 |  |  |
| Hamamdzic et al 2002 | 6 |  |  |
| Marie et al 2015 | 6 |  |  |
| Mayes 1996 | 6 |  |  |
| Mayes 1999 | 6 |  |  |
| McCormic et al 2009 |  |  | 6 |
| Mora 2009 | 5 |  |  |
| Nikpour et al 2010 | 6 |  |  |
| Radić et al. 2010 |  | 3 |  |
| Silman 1991 | 5 |  |  |
| Silman et al 1994 | 6 |  |  |
| Zhao et al 2016 |  |  | 7 |

^a^International Narrative Systematic Assessment; this scoring tool is used for the quality assessment of narrative reviews. ^b^National Heart, Blood and Lung Institute. INSA: International Narrative Systematic Assessment; NHLBI: National Heart, Blood, Lung Institute.
